# The superiority of innovative spiral-interdigital microelectrode pattern in increasing the sensitivity of tracing synchronization via serum starvation in cellular metabolism

**DOI:** 10.1038/s41598-024-68297-8

**Published:** 2024-08-03

**Authors:** Faegheh Bourbour, Mohammad Abdolahad, Fatemeh Hosseini Alast, Sogol Aslan Sefat

**Affiliations:** 1 Nano Electronic Center of Excellence, Nano Bio Electronic Devices Lab, School of Electrical and Computer Engineering, College of Engineering, University of Tehran, P.O.Box. 14395515, Tehran, Iran; 2 Department of Physics, Payame Noor University (PNU), P.O.Box 19395-4697, Tehran, Iran; 3 Nano Electronic Center of Excellence, Thin Film and Nano Electronics Lab, School of Electrical and Computer Engineering, University of Tehran, Tehran, Iran; 4 UT and TUMS Cancer Electronics Research Center, Tehran University of Medical Sciences, Tehran, Iran; 5 Nano Bio Electronic Devices Lab, Cancer Electronic Research Group, School of Electrical and Computer Engineering, College of Engineering, University of Tehran, Tehran, Iran; 6 Institute of Cancer, Imam Khomeini Hospital, Tehran University of Medical Sciences, Tehran, Iran; 7 Department of Physics, Shahid Beheshti University, Tehran, 19839 Iran

**Keywords:** Cell secretion, Microelectrode pattern, Cyclic voltammogram, Cell culture solution, MDA-MB-231 cell line, Cell synchronization, Cell starvation, Design, synthesis and processing, Electrical and electronic engineering, Lab-on-a-chip

## Abstract

In order to investigate the changes in the properties of the cell culture solution in the effect of cell synchronization via cell starvation (for 12, 24, and 36 h), a new spiral-interdigital pattern of microelectrode as a biosensor has been proposed. Then, to test its superiority, the results of this spiral-interdigital pattern with the results of the commercial pattern have been compared. The cells were selected from breast cancer standard lines (MDA-MB-231). Changes in CV peaks of the secretions were recorded by the spiral-interdigital pattern, in which increasing the interactive surface with homogenous electric paths had been considered by simulation before fabrication. The results of the simulation and experimental procedures showed a meaningful correlation. The occurrence of CV oxidative peaks at about 0.1–0.4 V and reductive peaks at approximately 0 V in the spiral-interdigital biosensor in the starved MDA-MB-231 cell line has been observed. The starvation situation resembles one that does not cause meaningful cell apoptosis or necrosis, and this method is only used to make the cells synchronized. Also, no peak is observed in normal cell growth conditions. In addition, by using the commercial design of the electrodes, no peak is observed in any of the conditions of normal and synchronized growth of the cells. Therefore, it seems that the observed peaks are caused by the agents that are secreted in the cell culture solution in a synchronized situation. Moreover, the design of the new spiral-interdigital electrode can significantly increase the sensitivity of the sensor to receive these peaks due to more space and a uniform electric field.

## Introduction

Due to their ease of use, non-invasiveness, and ability to extract valuable information, electrochemical methods are widely used in biological research^1^. On the other hand, cell secretion in its microenvironment can be affected by the cell's biological and growing states^2^. So, the cell microenvironment can contain valuable information about the cell conditions, which can be extracted in various ways, including electrochemical methods. Meanwhile, the cyclic voltametric (CV) method has been widely used as an electrochemical approach for tracing the different cellular condition cues^3^. In electrochemical methods such as CV, some of the substances in the cell culture solution can produce responses that can trace these changes as a result of applying an electric signal to the solution. In this regard, many studies have been established to obtain lots of information about tracing the condition of cells^4–8^. Furthermore, the careful examination of the materials and contents of the cell culture solution is very expensive and includes a complete examination of the pathway, complex proteomics, and molecular experiments to show the different chemical contents of the solution. So, the use of the CV method as an electrochemical method can be considered not only as complementary but also as a simple and useful method along with more complex methods.

**Figure 1:**
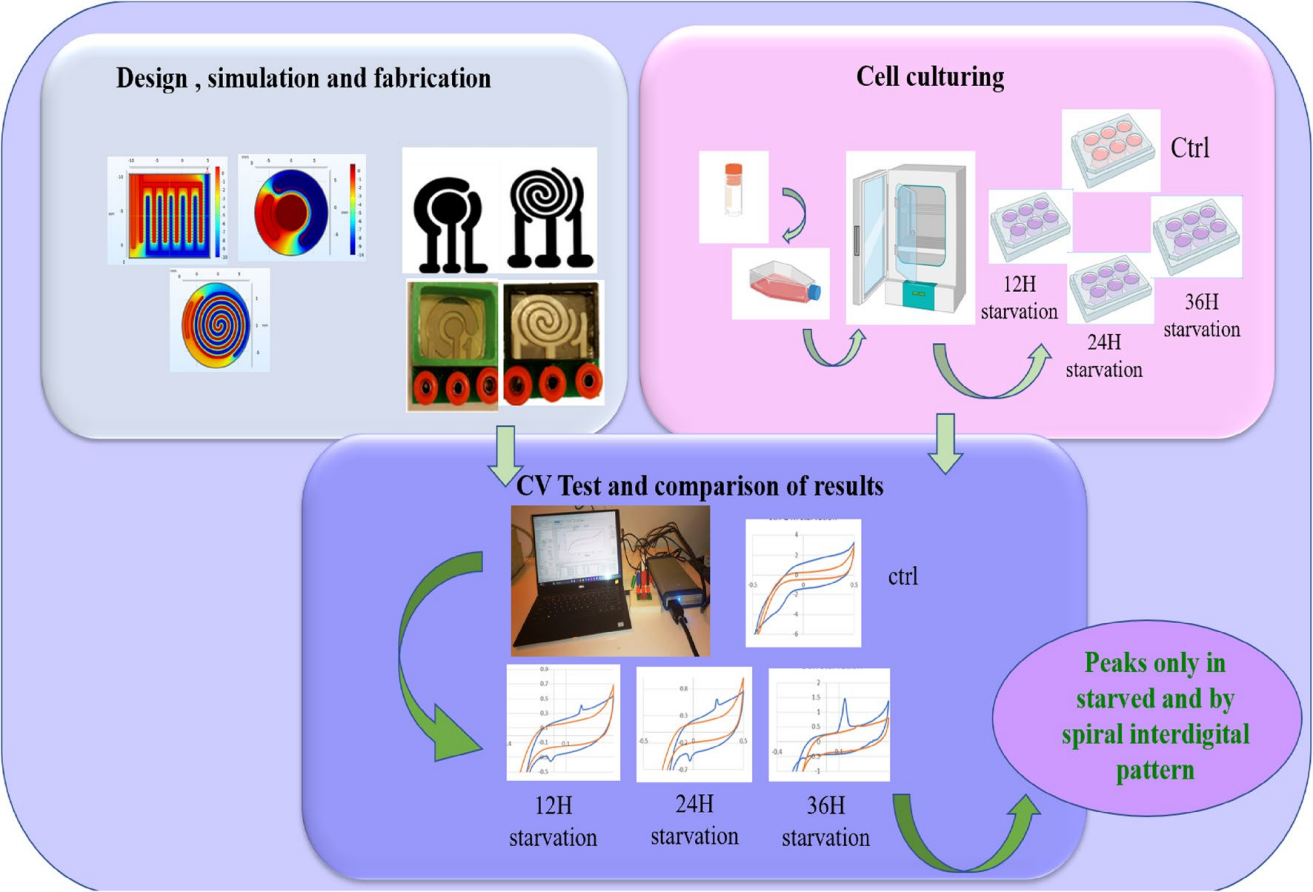
Graphical abstract of the complete steps of performing the test.

Moreover, cells go through the process of growth and division in a cycle called the cell cycle. This cycle includes the M, G1, S, and G2 phases. In a cell colony, the number of cells in every phase of this cycle is similar. Various methods, such as contact inhibition, centrifugal elutriation, and serum deprivation, can be used to synchronize them and increase the number of cells in a specific phase of the cell cycle^9–11^. In this research, the serum deprivation method has been used as the method of cell synchronization. The process of this method that has been used in this research will be explained in the following sections and the schematic of the steps carried out in this research has been shown in Fig. 1.

As it is clear, the duration of the phases of the cell cycle is different, and the dependence on serum and the nutrients in it is different for every cell type. So, the response of equal serum starvation duration time for different cell types will be different. In addition, the secretion of the cells in different phases of the cell cycle is different. Therefore, the method of serum starvation for cell synchronization and maybe for cell differentiation based on the constituents of the cell culture solution is an appropriate choice.

To perform CV tests, a gold-coated microelectrode with a fiberglass substrate and three electrodes for work, reference, and counter have been designed and used. Different designs are used for this purpose. Here, a design that is a combination of common and interdigital design has been used due to the advantages of both designs. With more space and a uniform electric field in the new chip design, optimal results for electrochemical tests are created, and the sensitivity of the CV method to detect the new peaks is increased.

This research may shed new light on cell-free monitoring of cellular metabolism and early warning about any problems that may happen in cellular incubation, which may cause changes in cell secretion without direct intervention on the cells or using flow cytometry.

## Materials and methods

### Device fabrication

**Figure 2:**
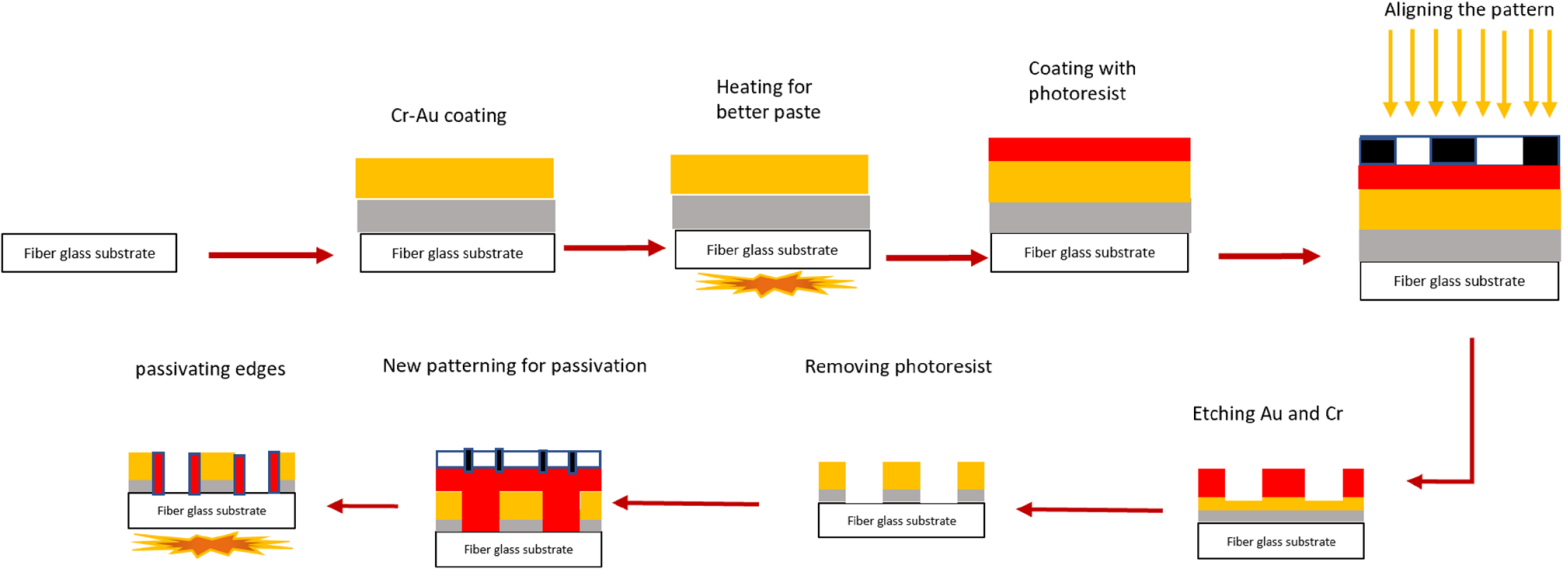
A schematic of the details of chip fabrication.

According to our previously published article^1^, one of the problems of microelectrodes with a glass substrate and gold coating is that due to the low adhesion of gold (despite the use of an intermediate layer of chromium) on the glass surface, with the increase in current passing from about 1.5 milliamperes, it causes the gold layer to peel off from the glass substrate and damage the sensor. In this research, we replaced the glass substrate with a fiberglass substrate and made some changes in the microelectrode fabrication steps in order to reduce this problem. Figure 2 depicts the schematic of these lithography process steps. The fabrication process started by coating a 1.54-mm fiberglass slide with 60 nm Cr and then 100 nm Au by RF sputtering (VAS Co.). The base pressure for the sputter deposition was adjusted to 5 × 10^–6^ mBar. After obtaining the base pressure, argon plasma was utilized to commence the deposition. The deposition pressure and power were adjusted to 2.5 × 10^–2^ mBar (100 W) and 1.8 × 10^–2^ mBar (250 W) for Cr and Au deposition, respectively. The thickness of the deposited layers was monitored during the deposition process using piezoelectric crystal oscillators inside the chamber. After the completion of the deposition process, in order to improve the adhesion of the Au and Cr layers to the fiberglass substrate, the fiberglass substrate has been heated for 45 min at 90 °C. After preparing the required layers, a positive photoresist (Shipley s1813) was spin-coated on the slide's surface for 30 s at 3000 RPM. Then, the slides were prebaked for 7 min at 90 °C before exposure. The alignment and exposure processes were performed using a mask aligner (Karl Suss MA6 mask aligner). After the exposure, the slides were developed and then post-baked for 4 min at 110 °C. Finally, the patterning process was completed by performing wet etching of the Au and Cr layers. To electrically passivate Au/Cr edges, a positive photoresist was spin-coated on the slide's surface for 30 s at 3000 RPM, one more time. Then, the slides were prebaked for 7 min at 90 °C before exposure.

The alignment and exposure processes were performed using a mask aligner in the reverse pattern with 0.5 mm more margin to cover edges. After the exposure, the slides were developed and then post-baked for 90 min at 180 °C. Here, it seems useful to mention this point: before performing the passivation steps, the surface of the slide has been washed with isopropyl alcohol.

### Precleaning of the device before the test

Before every test, the device has been rinsed with DI water, and the container has been filled up to 70% volume with DI water. Then, the chip surface was cleaned with a mild ultrasonic wave of 5-W power with its tip 5mm above the chip surface for 1 min to remove any residue that may have remained from the previous test^1^.

### Cell culture

MDA-MB-231, a human metastatic breast cancer cell line, was purchased from the National Cell Bank of the Pasteur Institute of Iran (NCBI). The considered cell lines (MDA-MB-231) were thawed in a cell culture flask. defreeze the desired cell (MDA-MB-231) using the relevant protocols and culture it in a 25 ml flask containing 89% Dulbecco's modified eagle medium (DMEM, Gibco) complemented with 10% fetal bovine serum (FBS, Bioceramed) and 1% penicillin/streptomycin (Bioceramed. It has been kept in in the normal situation in a CO2 incubator at 37 °C with 97% humidity and 5% CO_2_ for 5 days. After 5 days and when the confluency of the cells has reached about 80%, the cells of the flask have been collected. And 4 ml of 5% FBS, 1% penstrep and 94% DMEM culture solution were poured into each well of 2 six-well plates. Then about 20,000 cells were poured into each well. A manual cell counting method using a hemocytometer Neubauer has been used to measure the quantity of the cells in the flask and wells of the petri dish. After about three days passed and the filling of the cells reached about 50%, we collected the culture solution of the cells.

The cell culture solution from 9 of the six-well plates has been collected and replaced with 0.1% FBS, 1% penstrep and 99% DMEM culture solution after washing with phosphate buffered saline (PBS). After 12 h of serum deprivation, we collected the first three cells from plate number 1 and its corresponding control well and took their CV test. After 24 h of serum deprivation, this time collect the culture solution of the second three wells from the first plate and its corresponding control well and take their CV test. After 36 h of serum restriction, this time we collected the culture solution from the first three wells of the second plate and its corresponding control well and took their CV test. At last, we took a CV test of the wells with a normal cell culture solution. The condition of cells at the time of the normal culturing test has been shown in Fig. 9e.

### Viability test

In order to test the viability of cells, Annexin V/PI flow cytometry was performed. As we know, the rate of AnnexinV and PI absorption by the cells is related to the stage of their viability^12,13^. The test was done for the cell line (MDA-MB-231) that was in the normal culturing situation and also for the cells that had been kept in the situation of serum starvation for 12, 24, and 36 h. The results of these tests have been shown in Fig. 10 and Table 3.

### Cell cycle test

To find the presence percentage of cells in different phases of the cell cycle and to test the level of cell synchronization through the serum starvation method, flow cytometry tests were performed^14–16^, and the results are shown in Figs. 8, 9.

### Cyclic voltammetry

The CV method is increasingly used in the fields of biology due to its advantages, such as the non-invasive nature of the process and quick response, while at the same time containing valuable information about the tested solutions^17–21^.

In order to obtain electrochemical characteristics of cellular secretion, after accomplishing the cell culturing procedure as explained in section (2–1), 2 ml of the cell culture medium of each cell line (neither diluted nor pre-processed) were poured on the sensor, and the voltammogram was measured using both sensors. The response was measured using a portable electrochemical analyzer (IVIUM, Compact Stat. H) in three-electrode cyclic voltammetry (CV) mode^1^. The measurement was performed with a DC sweeping voltage of − 800 mV to 800 mV, and the scan rate was set to 50 mV/s. The results of these tests have been shown in Fig. 11.

### Ethical statement

All procedures performed in studies were in accordance with the ethical standards of the institutional and national research committee. This article does not contain any studies with animals performed by any of the authors.

## Results and discussions

To test the effect of cell synchronization via serum starvation on cell secretion, we designed a cyclic voltammetry electrochemical sensor with the combination of two common electrode patterns (commercial CV electrodes and spiral-interdigital electrodes). Their fabrication process was described in the “Materials and methods” section and the schematic of these steps is shown in Fig. 2.

**Figure 3:**
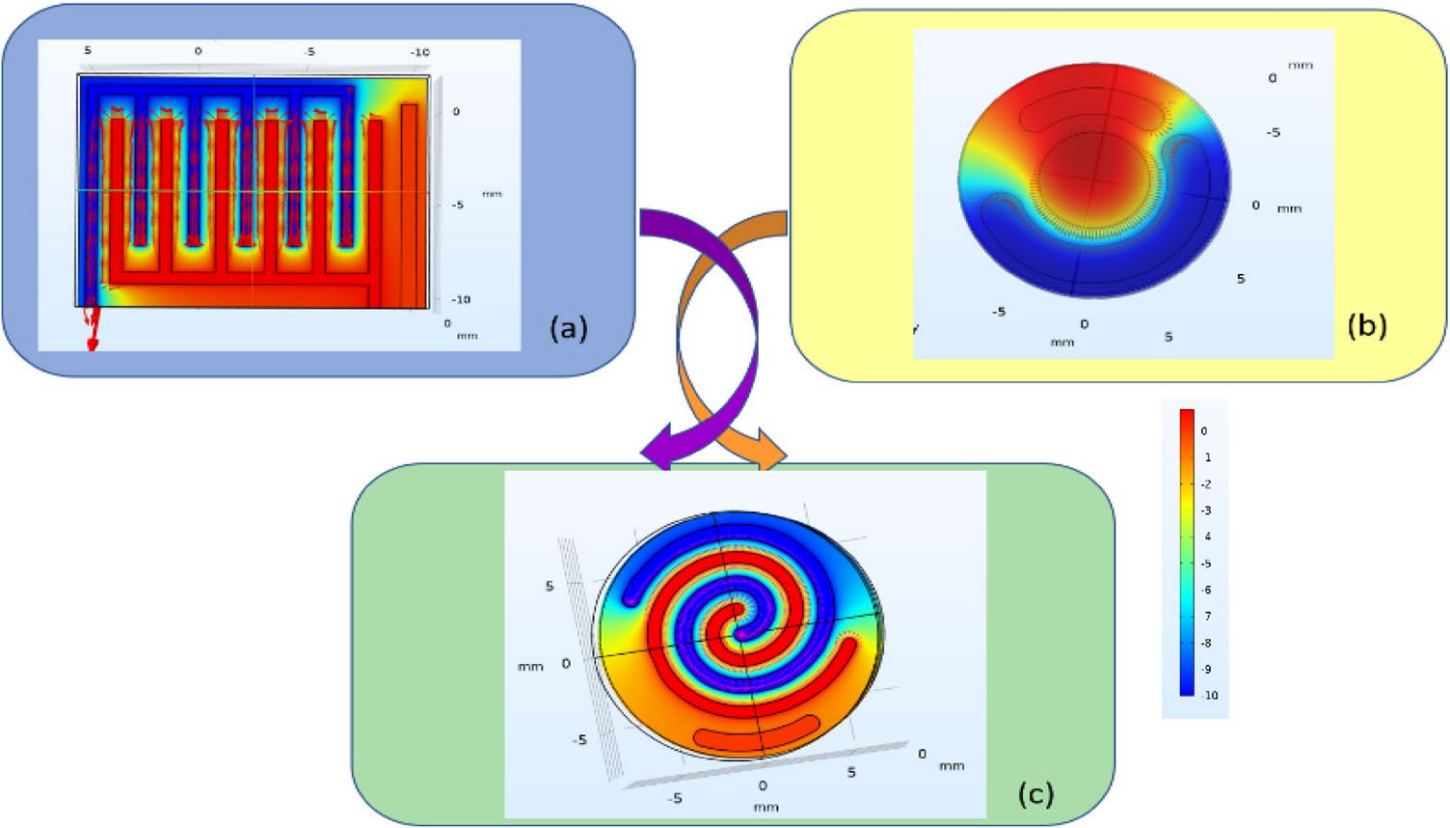
Simulation of electrical fields of (**a**) interdigital, (**b**) commercial, and (**c**) spiral-interdigital design by COMSOL Multi-physics Software.

The apparent differences between the electrical field distribution of these three patterns are shown in Fig. 3. The advantage of the new pattern is to increase the surface area with a uniform field and also to eliminate the edge effect and the problems caused by it. As described below with details about the reasons for the improvement of the performance of this new pattern, the results of the relevant CV test and the improvement of the sensitivity in receiving the electrochemical characteristics of the cell culture solution is shown in the following sections.

As expected, due to the increase of the surface with uniform electric field in the new pattern, we witnessed the improvement of performance and sensitivity of it and one of the results of which is stated in this research. COMSOL Multi-physics Software was used to show the electric field distribution in each of these designs and the results are shown in Fig. 3.

Some of the reasons that the spiral-interdigital pattern has better performance compared to other shapes of electrodes are as follows:

### Smoother current path

The spiral-interdigital geometry offers a continuous and smoother current path compared to the interdigital and commercial geometry. according to Fig. 3 and simulating the conditions and as expected from the spiral conditions, the field lines in this design are relatively parallel and uniform lines compared to other designs, and since the main factor in electrical current is the E-field distribution and path, It seems that these conditions can help to prove the uniformity of the current in this design compared to other designs. Using the results obtained in this article as well as the peaks observed in Fig. 11, this issue can be clarified more.

This promotes a more uniform distribution of the current across the electrode surface, reducing localized variations in the current density. As a result, the circular spiral electrode can provide more consistent and reliable measurements, leading to higher sensitivity.

### Reduction of edge effects

The edges of electrodes can introduce non-uniform current distributions and electric field gradients. In the case of a spiral-interdigital electrode, the absence of sharp corners or edges reduces the edge effects, resulting in a more homogeneous electric field distribution. This uniformity enhances the interaction between the electrode and the analyte, leading to improved sensitivity.

### Symmetry and homogeneity

Spiral-interdigital electrodes possess inherent symmetry and homogeneity due to their rotational symmetry. This symmetry can help maintain a uniform response across the electrode surface and minimize any spatial variations in sensitivity. In contrast, the interdigital electrodes may exhibit greater sensitivity variations due to the presence of corners and edges.

Now we want to discuss how, by removing the edge in a spiral shape and considering the other factors as constant, more sensitivity in our sensor has been achieved. According to the Nernst-Planck equation, we are going to prove this.

One of the key factors that influences mass transport in an electrochemical system is the migration and diffusion of charged species (ions) towards the electrode surface. The Nernst-Planck equation describes the flux of ions through a solution under the influence of an electric field and concentration gradients. The equation can be written as:

1 $$J=-D(dC/dx)+z*F*\upmu *C*E$$

In the Nernst-Planck equation (above), J stands for the ion flux (mol/m^2^s), D is the diffusion coefficient of the ion (m^2^/s), C is the concentration of the ion (mol/m^3^), x is the spatial coordinate (m), z is the charge number of the ion (dimensionless), F is Faraday's constant (C/mol), μ is the ion mobility (m^2^/Vs), and E is the electric field (V/m). In the equation, the first term on the right-hand side represents diffusion, while the second term represents migration due to the electric field. The uniform electric field (E) promotes a more uniform distribution of charged species, resulting in a more efficient mass transport of the analyte towards the electrode surface.

In the Nernst-Planck equation, the second term on the right-hand side represents the contribution of the electric field to the ion flux. When the electric field is uniform, it promotes a more uniform distribution of charged species (ions) in the solution. This uniform distribution reduces concentration gradients and enhances the migration of ions towards the electrode surface.

By reducing concentration gradients, a uniform electric field can facilitate the more efficient diffusion of ions towards the electrode, resulting in a higher ion flux. When the ion flux is higher, it can lead to enhanced sensitivity in certain electrochemical applications, such as ion-selective electrodes or electrochemical sensors.

However, it's important to note that the Nernst-Planck equation primarily describes the flux of ions in an electrochemical system and does not directly quantify sensitivity. Sensitivity is typically evaluated through experimental measurements, and it depends on various factors beyond mass transport, including the specific sensor design, analyte interactions, and detection techniques.

Therefore, while a uniform electric field can indirectly contribute to a higher ion flux by facilitating more efficient mass transport, it is just one factor among many that collectively determines the sensitivity of an electrochemical system or sensor.

### *Faradaic currents*^2^

In faradaic processes, a smoother current path can enhance the sensitivity by promoting a more uniform distribution of reactants and products at the electrode surface. When the current path is smooth, there are fewer localized areas of high current density or concentration gradients, which can lead to uneven reaction rates or non-uniform coverage of reactants. By ensuring a more uniform current path, the electrode surface experiences a more homogeneous distribution of the reactants, facilitating more efficient and reliable faradaic reactions. This can result in improved sensitivity for electrochemical sensing or other applications involving faradaic processes.

### Non-faradaic currents

In the case of non-faradaic currents, a smoother current path can also have implications. Non-faradaic currents often involve capacitive charging and discharging processes, such as in the case of double-layer capacitance at the electrode–electrolyte interface. Double-layer capacitance is a fundamental concept in electrochemistry that describes the capacitive behavior at the interface between an electrode and an electrolyte solution. A smoother current path can help reduce edge effects and non-uniform charge distribution, leading to a more uniform charging and discharging process. This can improve the stability and accuracy of non-faradaic current measurements and reduce artifacts or noise in the recorded signals.

### Electrode geometry

The geometry of the electrode plays a crucial role in determining the capacitance behavior. A smoother current path is often achieved by optimizing the electrode geometry to minimize irregularities, sharp edges, or surface roughness. By having a smooth and well-defined electrode surface, the electric field distribution becomes more uniform, reducing electric field gradients and non-uniform charge accumulation. This results in a more efficient charge storage process and enhanced double-layer capacitance.

### Electrolyte access

A smoother current path facilitates better access of the electrolyte to the electrode surface. Irregularities or roughness in the electrode surface can create regions where the electrolyte has limited access, leading to reduced charge storage capacity. A smoother current path ensures a more uniform flow of the electrolyte, allowing it to reach all areas of the electrode surface. This improved electrolyte access enhances the charge storage process and contributes to a higher double layer capacitance.

### Stability and reproducibility

A smoother current path improves the stability and reproducibility of the double-layer capacitance measurements. Irregularities or sharp edges in the electrode surface can lead to fluctuations or instabilities in the capacitance response. By achieving a smoother current path, these instabilities are minimized, resulting in more reliable and consistent capacitance measurements. This is particularly important in applications where accurate and repeatable capacitance values are required, such as in the characterization of energy storage devices or the evaluation of electrode performance.

**Figure 4:**
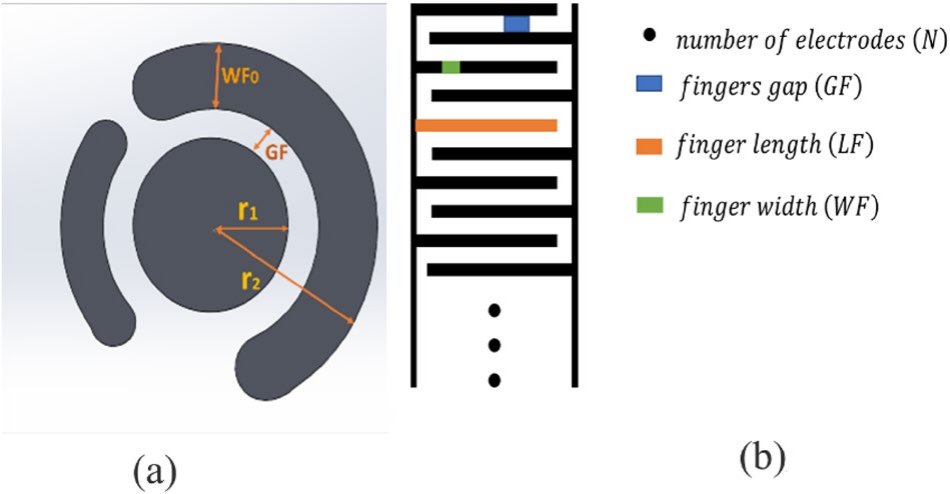
The variables needed to calculate the equivalent capacitance for (**a**) commercial and (**b**) interdigital pattern.

On the other hand, according to^22–24^, by using mathematical relationships, the reason for the increase in the sensitivity value in the interdigital spiral pattern can be proved as follows:

According to^24^ we can modulate interdigital sensor with some capacitance and our total capacity calculate from the following formula:

2 $${C}_{c}=4{\varepsilon }_{0}{\varepsilon }_{sm}^{*}\left(\frac{K\left({k}{\prime}\right)}{K\left(k\right)}\right),$$

3 $$K=\left(1+\frac{2\times WF}{2\times GF+WF}\right)\times \left(\sqrt{\frac{1}{1+\frac{2\times WF}{GF}}}\right)$$

$${k}{\prime}=\sqrt{1+{k}^{2}}$$

4 $${C}_{IDC}=\left[{\varepsilon }_{sm}^{*}{\varepsilon }_{0}\left(\frac{1+{\varepsilon }_{sub}}{2}\right)\times \frac{K\left(\sqrt{1+{k}^{2}}\right)}{K\left(k\right)}+{\varepsilon }_{sub}{\varepsilon }_{0}\frac{WF}{GF}\right]\times \left(N-1\right)LF$$

where,${\varepsilon }_{sm}^{*}$ represents complex permittivity of sensing material present on electrodes (in case of air ${\varepsilon }_{sm}^{*}$= 1 and in case of water ${\varepsilon }_{sm}^{*}$=78.4), ${\varepsilon }_{0}$ represents effective permittivity of free space (${\varepsilon }_{0}$=8.854*10^*–12*^) F/m, ${\varepsilon }_{sub}$ represents substrate permittivity, K represents the first elliptical integral, $k$ represents the ratio of finger width and finger gap (*WF/GF*), *N* represents the number of electrodes, and *LF* represents the length of electrode finger. The schematic of the variables needed for calculating the equivalent capacitance of commercial and interdigital pattern, has been shown in Fig. 4.

And for commercial pattern we can calculate the conductance as:

5 $$WF=W{F}_{avrage}=\frac{W{F}_{0}+2{r}_{1}}{2}, LF=\frac{3}{4}\left(2\pi {(r}_{2}-WF/2)\right) , GF={r}_{2}-{r}_{1}-\text{WF}_{0}$$

6 $${C}_{comercial}=\left[{\varepsilon }_{sm}^{*}{\varepsilon }_{0}\left(\frac{1+{\varepsilon }_{sub}}{2}\right)\times \frac{K\left(\sqrt{1+{k}^{2}}\right)}{K\left(k\right)}+{\varepsilon }_{sub}{\varepsilon }_{0}\frac{WF}{GF}\right]\times LF$$

Also, we saw that we can calculate the total capacitance of Spiral-Interdigital (${C}_{SIC}$) pattern by the following equations:

7 $${C}_{SIC}=\left[{\varepsilon }_{sm}^{*}{\varepsilon }_{0}\left(\frac{1+{\varepsilon }_{sub}}{2}\right)\times \frac{K\left(\sqrt{1+{k}^{2}}\right)}{K\left(k\right)}+{\varepsilon }_{sub}{\varepsilon }_{0}\frac{WF}{GF}\right]\times LF, GF1\approx GF2$$

**Figure 5:**
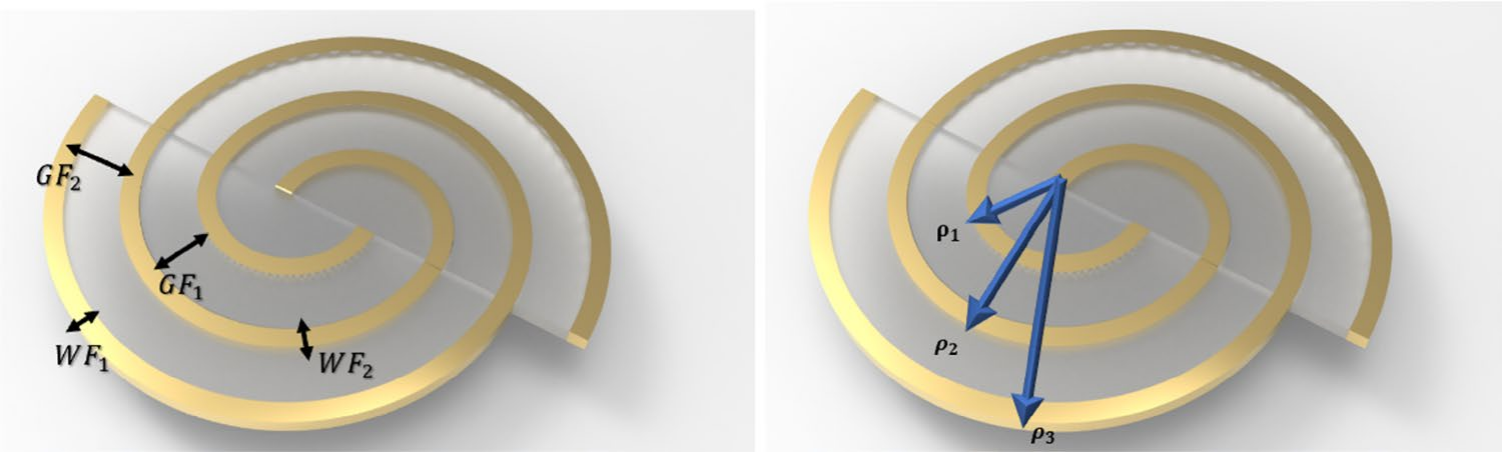
Schematic of the variables needed to calculate the equivalent capacitance for Spiral-interdigital pattern.

Now, to calculate the capacitance that we have in our substrate, which here is fiberglass, we can write it as the following formula, by using the value that we have in Table 1. the relevant parameters to calculate the equivalent capacitance of spiral-interdigital pattern has been shown in Fig. 5.

**Table 1:** Related variables of different patterns used for the calculation of their total capacities.

Variable	Description	Values of interdigit patern	Values of commercial pattern	Values of spiral interdigital patern
${x}_{av}$	Length of finger (LF)	8.3 (mm)	15.2 (mm)	40.53 (mm)
${a}_{av}$	Width of finger (WF)	0.7 (mm)	3.4 (mm)	1.25 (mm)
${d}_{av}$	Spacing between fingers (GF)	0.7 (mm)	1.5 (mm)	0.64 (mm)
$n$	No of fingers	6	2	2
$h$	Height of a substrate	1.54 (mm)	1.54 (mm)	1.54 (mm)
${\varepsilon }_{sub}$	Dielectric constant of a substrate	5.2	5.2	5.2
${\varepsilon }_{r}$’	Dielectric constant of a test sample	78.4	78.4	78.4

8 $$LF=L{F}_{avrage}=\pi \left({\rho }_{1}+{\rho }_{2}+{\rho }_{3}(3-8)+\frac{3}{2}\left(\frac{W{F}_{1}+W{F}_{2}}{2}\right)\right)$$

For calculating the values of capacitances, we had to calculate the values of K(k) and K(k’). So, we rewrite K as a function of k, and to drive this, we divide all the terms by GF, and we know that is equal to $WF/G$ .

Finally, by using the mentioned relations and by using the values of Table 1 for all three above-mentioned patterns, the values related to the total capacitors were obtained as follows:

total capacitor of *interdigital* pattern: 55 PF; total capacitor of *commercial* pattern: 171 PF; total capacitor of *spiral interdigital* pattern: 395.16 PF.

Since the relationships related to electrical sensitivity and impedance can be defined as follows:

9 $$S=\frac{\Delta {i}_{out}}{\Delta {v}_{in}}=\frac{1}{{z}_{in}}$$

10 $${z}_{in}=\frac{1}{j{C}_{total}W}+{R}_{total}$$

Here ${R}_{total}$ is similar for all three patterns; by increasing ${C}_{total}$ we have lower ${z}_{in}$ and higher sensitivity.

So, by removing the edge effect, we can reach a higher ion flux that causes an increase in sensitivity by using the equation of sensitivity mentioned above.

**Figure 6:**
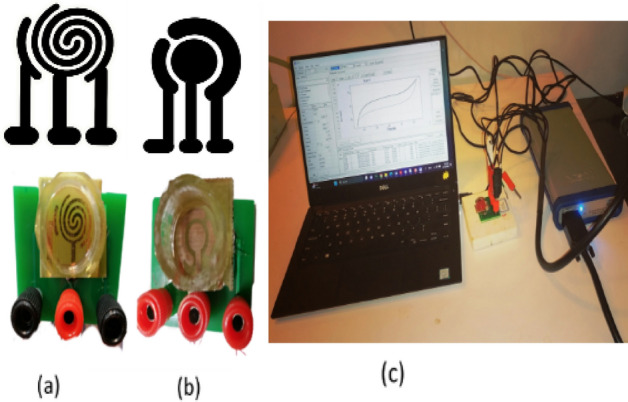
The last fabricated biosensors with (**a**) spiral-interdigital, (**b**) commercial patterns and (**c**) the last set up for CV test.

**Figure 7:**
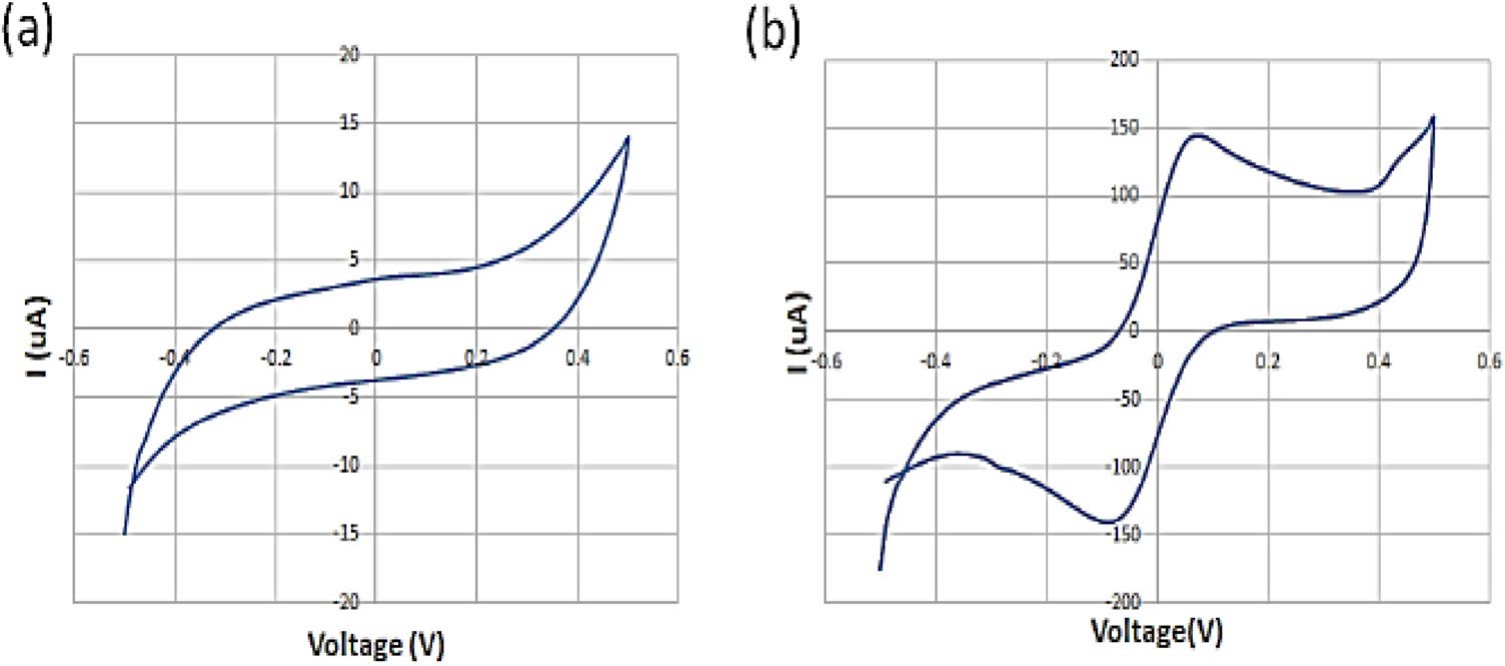
The obtained voltammogram of the (**a)** DI water, (b**)** 125 μM of standard redox probe (K3[Fe(CN)6]) by spiral interdigital pattern of microelectrode^1^.

The set-up used in this research was made in two commercial and spiral-interdigital patterns by three electrodes (reference, work, and counter), represented in Fig. 6. The smaller electrode is related to the reference, the middle electrode is related to the work, and the remaining electrode is related to the counter (Fig. 7).

**Figure 8:**
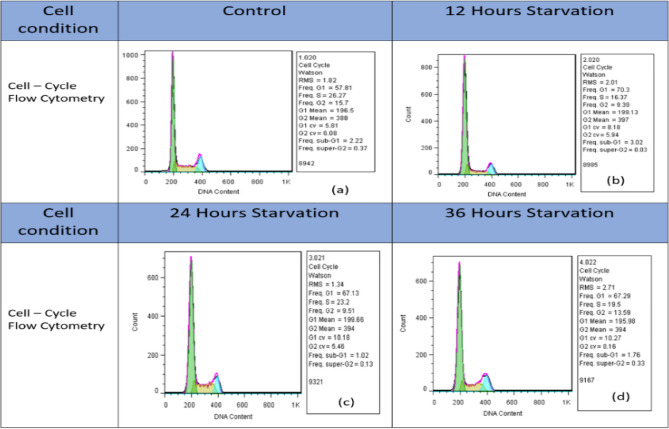
The presence of cells in different phases of the cell cycle according to the flow cytometry test for (**a**) Control (**b**) 12Hours Starvation (**c**) 24 Hours Starvation and (**d**) 36 Hours Starvation Conditions of MDA-MB-231 cell respectively and (**e**) condition of MDA-MB-231 cell line with 80% confluency in 25 ml flask.

**Figure 9:**
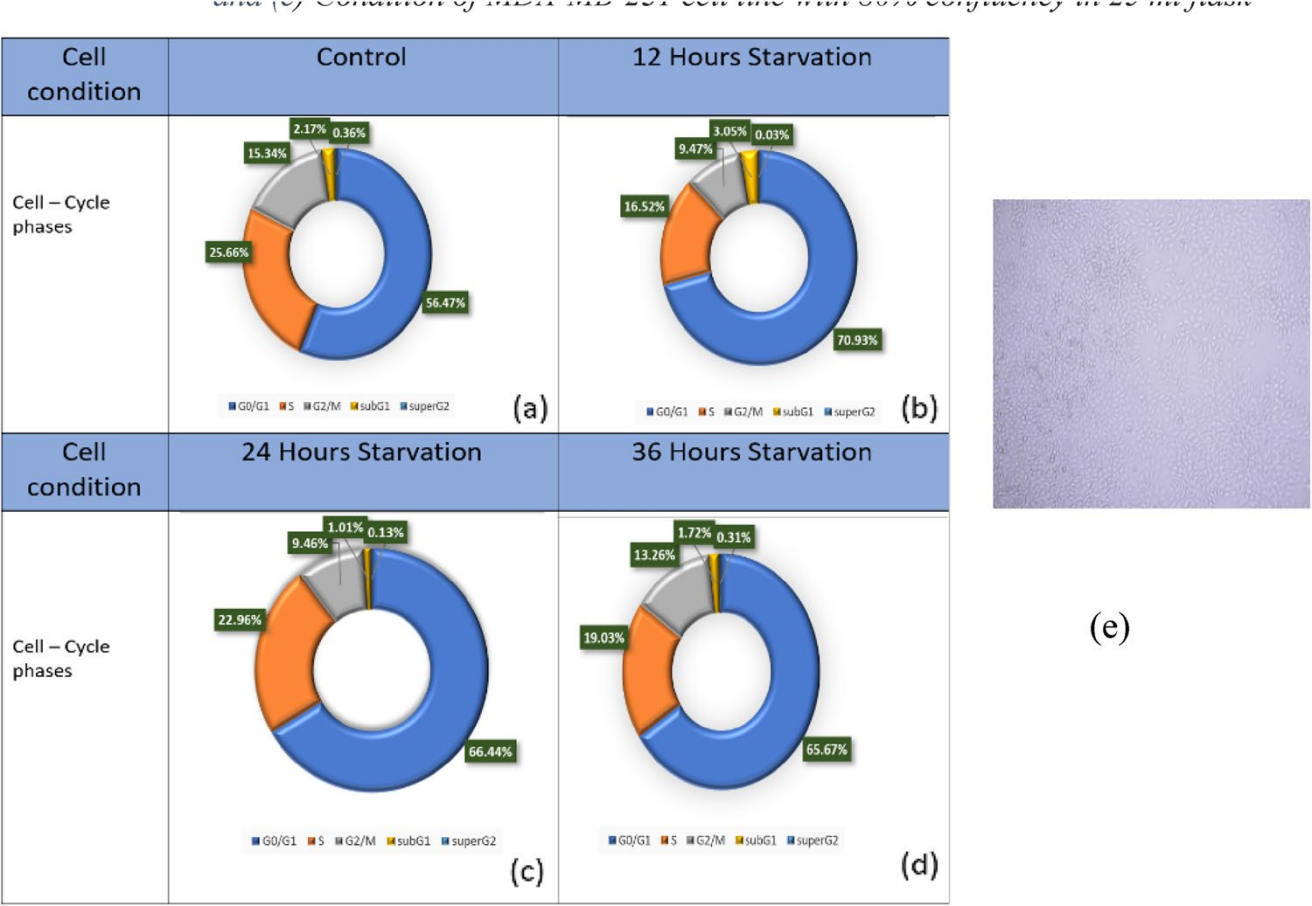
The Frequency of the presence of cells in different phases of the cell cycle according to the flow cytometry test for (**a**) Control (**b**) 12Hours Starvation (**c**) 24 Hours Starvation and (**d**) 36 Hours Starvation Conditions of MDA-MB-231 Cell respectively.

From the pieces of evidence, it seems that cells will secrete different materials in different situations. So, we can apply different conditions to the cells and test their responses to these conditions. In this research, in order to impose different conditions to test changes in cell secretions and then test these changes using the CV method, cell synchronization via serum starvation protocols was carried out. The results have been shown in Figs. 8, 9 and Table 2.

**Table 2:** Quantitative results of The Frequency of the presence of cells in different phases of the cell cycle according to the flow cytometry test For Control,12Hours Starvation, 24 Hours Starvation and 36 Hours Starvation Conditions of MDA-MB-231 Cell respectively.

Cell type	(G0/G1)	S	G2/M	Sub G1	Super G2
Control	57.81	26.27	15.7	2.22	0.37
12 h starvation	70	16.37	9.39	3.02	0.03
24 h starvation	67.13	23.2	9.51	1.02	0.13
36 h starvation	67.2	19.5	13.59	1.76	0.33

In order to investigate the response of the new pattern of microelectrode to the standard condition, the corresponding CV test was performed for DI water and a standard redox solution of 125 μM of K3[Fe(CN)6], and the results are shown in Fig. 7.

**Figure 10:**
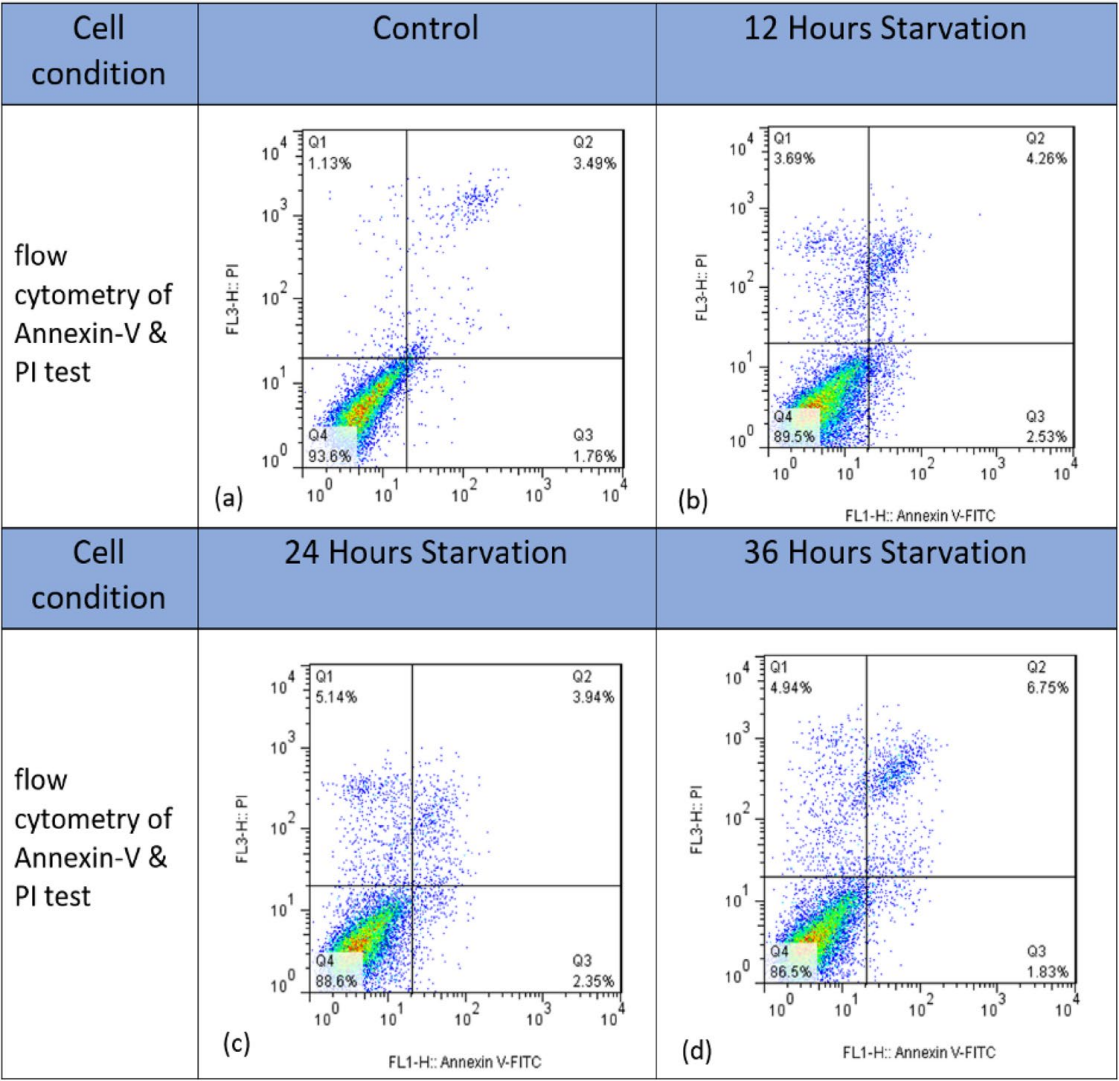
Results of flow cytometry of Annexin-V & PI test (**a**) Control (**b**) 12Hours (**c**) 24 Hours and (**d**) 36 Hours Starvation Conditions of MDA-MB-231 Cell respectively.

Next, flow cytometry was used to investigate the effect of starving on cell synchronization. As these results are shown in Fig. 10, it can be seen that, as expected, by removing the serum for 12, 24, and 36 h, the percentage of cells in the G0/G1 phase of the cell cycle increases by more than 10% compared to the control state. However, increasing the starving time does not show a significant effect on this amount.

In addition, in order to investigate the effect of serum removal for 12, 24 and 36 h on the cell viability, Annexin V and PI flow cytometry test was performed on these cells and the results can be seen in Fig. 9 and Table 2 As can be seen in the Fig. 10 and in the relevant Table 3, by removing the serum from the cells for 12, 24 and 36 h, the survival rate of the cells is also reduced between 4 and 7%, which can also be attributed to this phenomenon and the stressful conditions caused by it can also cause the secretion of substances in the cell culture solution that the peaks observed in the figure are also under their influence, and for a more accurate conclusion of these conditions, a more detailed investigation of the contents of the culture solution is needed.

**Table 3:** Quantitative analysis of the Annexin V and PI test for Control, 12Hours, 24 Hours and 36 Hours Starvation Conditions of MDA-MB-231 Cell.

Cell condition	Live%	Early apoptosis%	Late apoptosis%	Necrosis%
Control	93.6	1.76	3.49	1.13
12 h starvation	89.5	2.53	4.26	3.69
24 h starvation	88.6	2.35	3.94	5.14
36 h starvation	86.5	1.83	6.75	4.94

**Figure 11:**
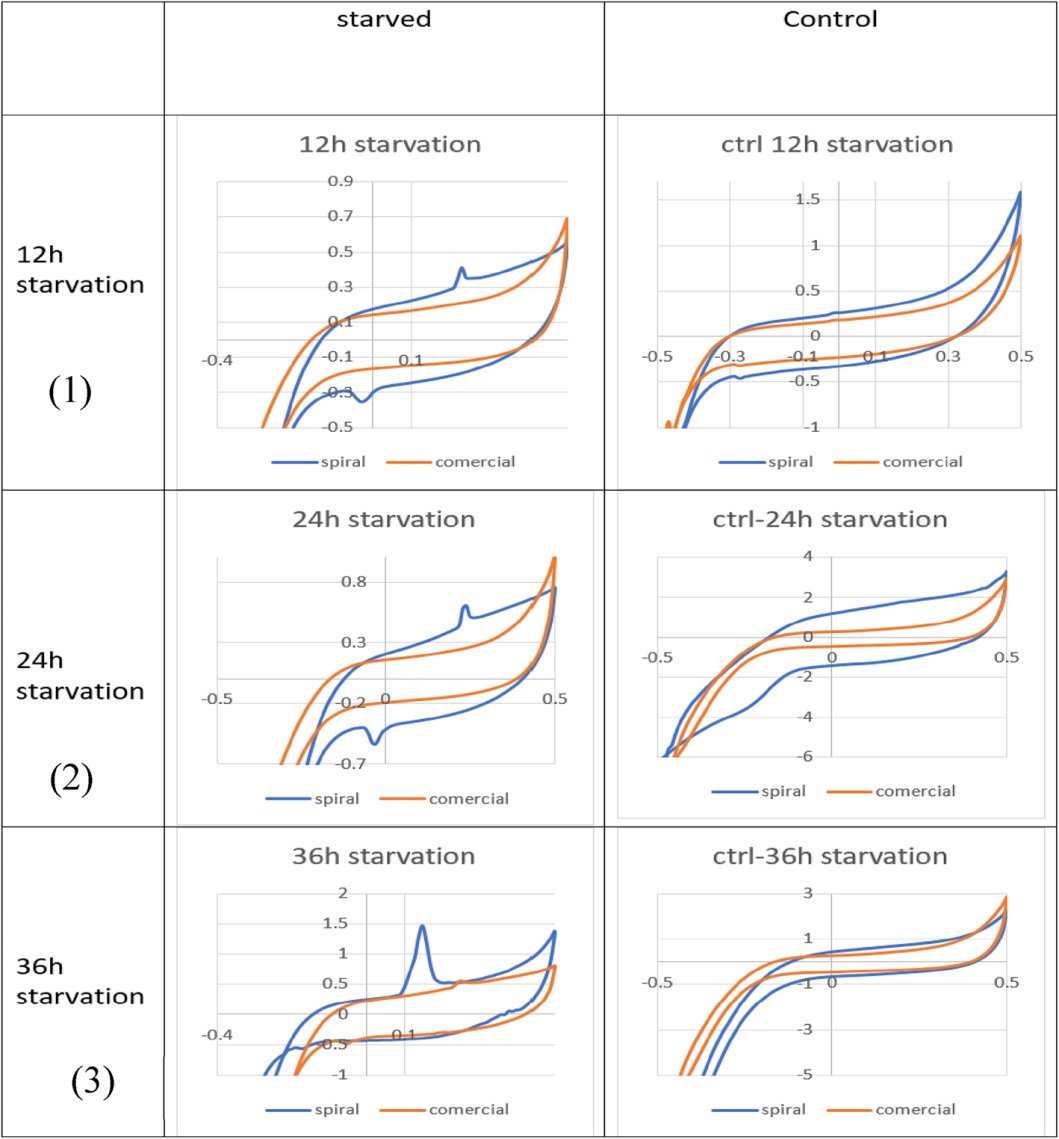
Comparison of cyclic voltammograms (CV) of cell-free cell culture solution of (**a**) starved and (**b**) Normal culturing situation of (1) 12 Hours, (2) 24 Hours and (3) 36 Hours of serum starvation by spiral-interdigital (blue line) and commercial (orange line) pattern chips respectively.

According to the results shown in Figs. 8, 9 and Table 2, it seems that by removing the cell serum for 12, 24, and 36 h, the cells become synchronized to some extent, and as a result of this synchronization of the cells or other factors that require further investigation of the contents and conditions of a cell, the cells are under stress conditions, and as a result of these conditions, they have secreted substances into the culture solution, and these substances can be seen in the form of peaks seen in Fig. 11.

The remarkable point is that, due to the increased sensitivity of the microelectrode with the interdigital spiral pattern, these peaks can only be seen in this design and are not visible in the commercial design.

At last, the results of the CV test of starved cells for 12, 24, and 36 h and their related control cells are shown in Fig. 11. As can be seen in Fig. 11, in the condition of 12 h of starvation, the voltage of the oxidation peak occurs between 0 and 0.25 V, and their related currents are between 0.35 and 0.4 mA. The voltage of the reduction peak occurs between 0 and − 0.2 V, and the related current is about − 0.35 mA. In the condition of 24-h starvation, the voltage of the oxidation peak occurs at about 0.2 V, and their related currents are between 0.35 and 0.4 mA. The voltage of the reduction peak occurs at about 0 V, and the related current is between − 0.35 and − 0.5 mA. In the condition of 36 h of starvation, the voltage of the oxidation peak occurs between 0.15 and 0.25 V, and their related currents are between 0.5 and 1.5 mA. The voltage of the reduction peak occurs between − 0.18 and 0 V, and the related current is about − 0.5 mA.

## Conclusion

As we discussed in the topics raised in this research, it can be concluded that one of the conditions that can be used to increase the sensitivity of sensors is the use of patterns that are used in microelectrodes. This can be achieved by using designs that provide the effective interaction surface of the biosensor with the analytes with a uniform field. In addition, as we discussed before, it can be investigated by creating different stress conditions, such as serum starvation and its effects on the cells, which leads to synchronization of the cell and possibly other unknown side effects, and by examining the electrochemical properties of the cell culture solution. Other contents of the cell culture solution as well as the conditions of the cells achieved effective and valuable results in relation to the cell characteristics.

Also, these results can be done for different cells or at different intervals of serum starvation, and the results can be compared with each other. These results can be used to identify the different characteristics of cells and possibly to diagnose the characteristics of cancer cells.

In addition to the above, it is possible to use similar three-dimensional designs for increasing the sensitivity of biosensors and for cells with a fibroblastic structure. Also, the use of materials with a higher interaction level and the use of functional agents were also used to improve sensitivity.

## Data Availability

All data generated or analyzed during this study are included in this published article.
